# Psychedelics in developmental stuttering to modulate brain functioning: a new therapeutic perspective?

**DOI:** 10.3389/fnhum.2024.1402549

**Published:** 2024-06-19

**Authors:** Giuseppe Pasculli, Pierpaolo Busan, Eric S. Jackson, Per A. Alm, Danilo De Gregorio, Gerald A. Maguire, Guy M. Goodwin, Gabriella Gobbi, David Erritzoe, Robin L. Carhart-Harris

**Affiliations:** ^1^Department of Computer, Control, and Management Engineering (DIAG), La Sapienza University, Rome, Italy; ^2^Italian Society of Psychedelic Medicine (Società Italiana di Medicina Psichedelica–SIMePsi), Bari, Italy; ^3^IRCCS Ospedale San Camillo, Venice, Italy; ^4^Department of Communicative Sciences and Disorders, New York University, New York, NY, United States; ^5^Department of Public Health and Caring Sciences, Uppsala University, Uppsala, Sweden; ^6^IRCCS, San Raffaele Scientific Institute, Milan, Italy; ^7^Vita-Salute San Raffaele University, Milan, Italy; ^8^School of Medicine, American University of Health Sciences, Signal Hill, CA, United States; ^9^CenExel CIT Research, Riverside, CA, United States; ^10^Department of Psychiatry, University of Oxford, Oxford, United Kingdom; ^11^Neurobiological Psychiatry Unit, Department of Psychiatry, McGill University, Montreal, QC, Canada; ^12^Department of Medicine, Centre for Psychedelic Research, Imperial College London, London, United Kingdom; ^13^Psychedelics Division, Neuroscape, University of California, San Francisco, CA, United States

**Keywords:** developmental stuttering, psychedelic compounds, neuropsychopharmacology, default mode network, social-cognitive networks

## Abstract

Developmental stuttering (DS) is a neurodevelopmental speech-motor disorder characterized by symptoms such as blocks, repetitions, and prolongations. Persistent DS often has a significant negative impact on quality of life, and interventions for it have limited efficacy. Herein, we briefly review existing research on the neurophysiological underpinnings of DS -specifically, brain metabolic and default mode/social-cognitive networks (DMN/SCN) anomalies- arguing that psychedelic compounds might be considered and investigated (e.g., in randomized clinical trials) for treatment of DS. The neural background of DS is likely to be heterogeneous, and some contribution from genetically determinants of metabolic deficiencies in the basal ganglia and speech-motor cortical regions are thought to play a role in appearance of DS symptoms, which possibly results in a cascade of events contributing to impairments in speech-motor execution. In persistent DS, the difficulties of speech are often linked to a series of associated aspects such as social anxiety and social avoidance. In this context, the SCN and DMN (also influencing a series of fronto-parietal, somato-motor, and attentional networks) may have a role in worsening dysfluencies. Interestingly, brain metabolism and SCN/DMN connectivity can be modified by psychedelics, which have been shown to improve clinical evidence of some psychiatric conditions (e.g., depression, post-traumatic stress disorder, etc.) associated with psychological constructs such as rumination and social anxiety, which also tend to be present in persistent DS. To date, while there have been no controlled trials on the effects of psychedelics in DS, anecdotal evidence suggests that these agents may have beneficial effects on stuttering and its associated characteristics. We suggest that psychedelics warrant investigation in DS.

## Introduction

Developmental stuttering (DS), also known as Childhood-Onset Fluency Disorder (American Psychiatric Association, 2013), is a neurodevelopmental disturbance characterized by speech and motor symptoms including repetition, halting, and prolonging of syllables (Bloodstein et al., 2021). In addition, language processing may be affected to some extent (both in childhood and adulthood; e.g., Weber-Fox et al., 2013; Gastaldon et al., 2023). Thus, DS is a complex and multifactorial impairment where speech-motor difficulties often negatively impact the quality of life of persons who stutter (PWS), resulting in a series of associated symptoms including anxiety and social avoidance (especially in “persistent” DS; Menzies et al., 1999; Iverach et al., 2011; Iverach and Rapee, 2014). On the other hand, when stuttering begins in adulthood, it is usually the consequence of a brain injury (neurogenic stuttering; Junuzovic-Zunic et al., 2021). Recent work is clarifying the brain dynamics of DS using neuroimaging and neurophysiological methods: indeed, stuttering is thought to be a speech-motor issue related to basal ganglia dysfunction and impairments of speech/motor cortical areas, also resulting in atypical neural connectivity (Etchell et al., 2018). Thus, while there are many unanswered questions regarding the underlying neural basis of DS, a common perspective is that stuttering is associated with altered brain regulation of motor sequencing and timing of volitional control of speech (Craig-McQuaide et al., 2014; Etchell et al., 2014, 2015, 2018; Busan et al., 2019, 2020; Busan, 2020; Chang and Guenther, 2020; Tendera et al., 2020; Alm, 2021b; Gastaldon et al., 2023; Orpella et al., 2024). Nevertheless, stuttering remains difficult to treat and no resolutive treatment is available, especially when it persists in adulthood. Speech therapy is the most commonly used treatment, but relapse is common (Qureshi et al., 2021). Neuromodulation and pharmacology are also increasingly investigated (for recent reviews see Maguire et al., 2020; Busan et al., 2021; Qureshi et al., 2021; see also Chesters et al., 2018, Garnett et al., 2019; Busan et al., 2023). For example, experimental pharmacological interventions in DS modulate the (nigro-striatal) dopaminergic system of the brain, typically by means of dopamine antagonists (e.g., at D2 [risperidone or olanzapine] and D1 receptors [ecopipam]; Maguire et al., 2020). In this context, serotonergic (e.g., paroxetine) or GABAergic (e.g., pagoclone) interventions have also been investigated (Maguire et al., 2020).

Recently, psychedelic drugs (i.e., serotonergic agonists producing psychedelic effects) have sparked significant interest due to their potential therapeutic effects on psychiatric conditions such as depression, (social) anxiety, and post-traumatic stress disorder (PTSD; Luoma et al., 2020; Inserra et al., 2021a). Moreover, they are also under evaluation for movement disorders such as Parkinson’s disease (Wolff et al., 2023). Classic psychedelic drugs (e.g., psilocybin, lysergic acid diethylamide [LSD], and dimethyltryptamine [DMT]) have high affinity for the 5-HT2A serotonin receptor, but their mechanisms of action remain unclear and/or heterogenous (Moujaes et al., 2023). However, these drugs may be able to facilitate acute periods of psychological/cognitive flexibility that may promote neural plasticity that aids in changing behavioral “maladaptive” patterns (e.g., Carhart-Harris and Friston, 2019). In this regard, this perspective article puts forth the idea that psychedelic drugs might complement existing behavioral therapies, with potential fluency-facilitating effects and contributing to reduced psychosocial impact (e.g., anxiety and social avoidance), thus improving the quality of life of PWS (compare with Jackson et al., 2024). Herein, we will describe the (heterogeneous) neural and psychological background factors related to DS, as well as their possible interactions with mechanisms hypothesized for psychedelic drugs. Finally, a rationale for applying this class of drugs in DS will be proposed. For the sake of brevity, we do not make an overt distinction between data obtained from different cohorts (e.g., adult vs. children who stutter, human vs. animal models, clinical vs. pre-clinical data, different compounds, etc.) and the reader is addressed to additional references for further in-depth analyses.

## The neurophysiological basis of DS: brief overview of the current state

### Background factors

At the neurobiological level, two main themes emerge in the current research: (i) the possible role of dopamine in stuttering, and (ii) indications that stuttering may be related to impaired supply of energy to neurons (e.g., sub-optimal glycolysis processes; Wu et al., 1997; Alm, 2004, 2021a,b; Civier et al., 2013; Chang and Guenther, 2020; Chow et al., 2020; Maguire et al., 2021; Turk et al., 2021). It has been speculated that both these mechanisms might be related to genetically determined cellular metabolic limitations (especially in frontal lobe) that become evident during speech, since it may be the most energy-demanding motor task (Alm, 2021a; compare with Civier et al., 2013; Chang et al., 2019; Chang and Guenther, 2020). Cellular structures such as astrocytes and lysosomes can be also affected, thus playing a possible role in the etiopathogenesis of DS (e.g., Kang et al., 2010; Kang and Drayna, 2012; Han et al., 2019; Benito-Aragón et al., 2020; Chow et al., 2020; Maguire et al., 2021; Turk et al., 2021). Notably, studies such as that by Kraft and Yairi (2012) have highlighted a major genetic influence in the etiology of stuttering. The most compelling evidence so far points to a group of genes that are integral to intra-cellular transport and recycling processes in lysosomes, as elucidated by Barnes et al. (2016) and Frigerio-Domingues and Drayna (2017). Among these, *GNPTAB* and associated genes are instrumental in lysosomal function by affecting the trafficking of hydrolase enzymes to lysosomes. In fact, under normal conditions, *GNPTAB* is involved in synthesizing mannose 6-phosphate, a key element for directing hydrolase enzymes to lysosomes. Mutations in *GNPTAB* disrupt this essential process, thus leading to improper or insufficient delivery of these enzymes. This disruption may result in a marked deficiency of functional hydrolases in the lysosomes, thereby hampering the degradation of glycoproteins and glycolipids. The resultant accumulation of undegraded substrates in the lysosomes can in turn impact cellular functions and contribute to the pathophysiology of DS (Alm, 2021a). Moreover, mutations in the *GNPTAB* gene may result in a reduced number of astrocytes, especially when considering structures such as the corpus callosum, which is fundamental for inter-hemispheric communication (and, thus, for speech and/or vocalization; Han et al., 2019).

On the other hand, when it comes to non-genetic factors, a recent analysis has provided evidence that streptococcal infections appear to have been a major cause of stuttering before penicillin became available by triggering an autoimmune reaction that may have affected the basal ganglia (i.e., pediatric autoimmune neuropsychiatric disorders associated with streptococcal infections [PANDAS]; Alm, 2020). Nonetheless, it is important to emphasize that the stuttering group is likely to be heterogeneous from a neurobiological perspective (e.g., when considering individual differences in responses to dopaminergic drugs; Alm, 2004, section 6.1.2).

### Neurological mechanisms

**FIGURE 1:**
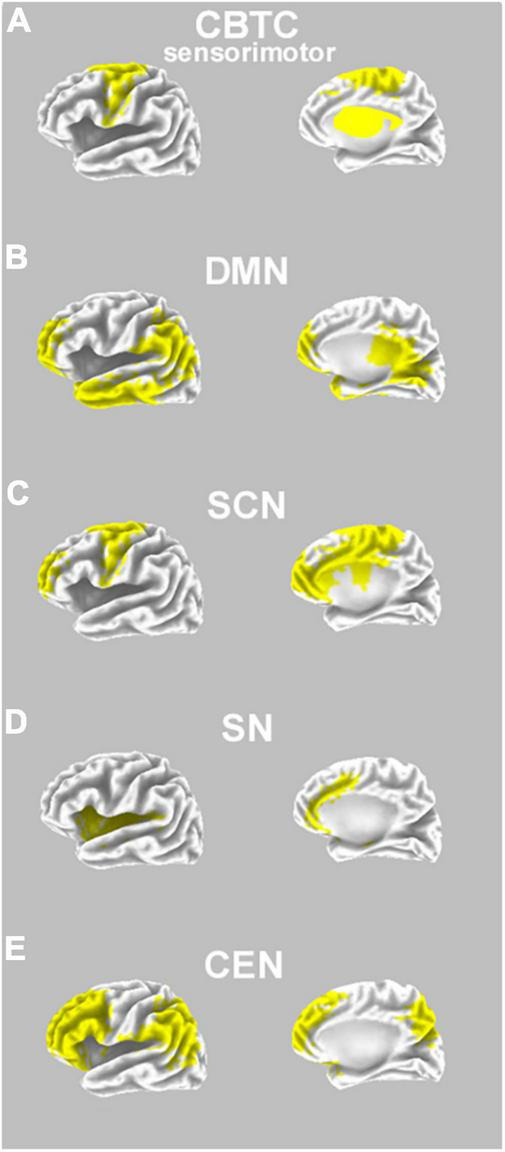
Neural pathways involved in DS, and possible interactions with psychedelics effects. **(A)** A prototypical representation of the (sensorimotor) cortico-basal-thalamo-cortical circuit (CBCT), which has a role in DS neuropathology, and includes regions such as the premotor cortex, supplementary motor area, thalamus, and basal ganglia (bilaterally, in yellow on an inflated model; note that basal ganglia and thalamus are schematically superimposed only for descriptive purposes); **(B)** a prototypical representation of the default mode network (DMN), hypothesized to have a role in DS neuropathology as well as hypothesized to be a primary neural target of psychedelics; DMN comprises regions such as the prefrontal cortex, temporal cortex, parietal cortex, and posterior cingulate cortex (bilaterally, in yellow on an inflated model); **(C)** a prototypical representation of a social-cognitive network (SCN; based on Amodio and Frith, 2006; compare with Alm, 2014), which has an influence on the symptoms of DS; SCN comprises prefrontal regions, also influencing functionality of the anterior cingulate cortex and the motor cortex (e.g., supplementary motor area; all regions are shown in yellow on an inflated model); **(D)** a prototypical representation of the salience network (SN), which is useful for switching from DMN to TPNs, and comprises bilateral regions such as the anterior cingulate cortex and insula (in yellow on an inflated model); **(E)** a prototypical representation of the central executive network (CEN; as part of task-positive networks, TPNs), comprising regions such as the dorsolateral prefrontal cortex and parietal cortex (bilaterally, in yellow on an inflated model).

From a neural point of view, many theories of stuttering focus on the role of motor timing or on other aspects of speech-motor sequencing (Van Riper, 1982; Alm, 2004, 2021b; Etchell et al., 2014, 2015; Busan et al., 2019, 2020; Busan, 2020; Chang and Guenther, 2020; Jenson et al., 2020; Tendera et al., 2020). As a corollary, DS seems to be a disorder characterized by impairments in cortico-basal-thalamo-cortical networks (crucial for implementing and executing voluntary motor sequences, such as those needed for speech; Alm, 2004; Civier et al., 2013; Craig-McQuaide et al., 2014; Etchell et al., 2014, 2015, 2018; Busan, 2020; Chang and Guenther, 2020; Figure 1A). Structural and functional abnormalities are evident in cortical and sub-cortical regions (and their connections) including the supplementary motor area, inferior frontal regions, temporal cortex, sensorimotor cortex, basal ganglia, and the connecting white matter tracts (Sommer et al., 2002; Watkins et al., 2008; Craig-McQuaide et al., 2014; Neef et al., 2015, 2016, 2018a,b, 2022, 2023; Kronfeld-Duenias et al., 2016; Etchell et al., 2018). In this context, a very recent meta-analysis confirmed that DS is characterized by structural/functional alterations in wider brain networks, including cortico-cortical and sub-cortical circuits, that support speech fluency and motor sequences (Matsuhashi et al., 2023). These alterations may differently sustain “trait” and “state” stuttering (Budde et al., 2014; Belyk et al., 2015, 2017; Connally et al., 2018).

### A role for the default mode network in DS?

Functionally, the brain of PWS is characterized by atypical neural activity during speech-motor programming and execution, but also during rest (for a recent review see Etchell et al., 2018). For example, using functional magnetic resonance imaging (fMRI) during resting conditions, Desai et al. (2017) reported reduced blood perfusion of speech-motor areas in DS. In another study, Xuan et al. (2012) suggested the presence of abnormal neural activity and functional connectivity in a series of resting-state networks, also comprising sensorimotor regions and nodes of the default mode network (DMN; Figure 1B). The DMN is a group of interconnected brain regions (mainly comprising nodes such as the prefrontal cortex, lateral/inferior parietal cortex, temporal cortex, and the posterior cingulate cortex; Andrews-Hanna et al., 2014) characterized by high basal metabolism, perfusion, small world architecture, and functional and geodesic distance from the “unimodal” cortex (Raichle, 2015). The DMN tends to show a dip in activity when the brain is actively involved in “goal-oriented” task execution (despite a growing amount of data highlighting a key role of the DMN in itinerant, semantically abstract, internally-directed and/or self-generated/self-referential cognition; Andrews-Hanna et al., 2014; Raichle, 2015). As a consequence, there is orthogonality (if not functional antagonism to the DMN) in the so-called task-positive networks (TPNs) that, for example, are activated during attention-demanding tasks in interaction with external cues (Raichle et al., 2001; Hamilton et al., 2011). In fact, the DMN has high basal activity but is typically most activated when attention is not directed toward an external stimulus (e.g., during excessive rumination, which is a common condition in anxiety and depressive disorders; Hamilton et al., 2011; Fernandes Coutinho et al., 2016). Interestingly, there are indications that the DMN has a role in speech disorders, such as the presence of higher connectivity between the DMN and fronto-parietal networks (Cai et al., 2024), as well as in DS pathophysiology (Chang et al., 2018; Garnett et al., 2022). More specifically, Chang et al. (2018) reported the presence of a resting-state aberrant network connectivity in PWS, involving DMN regions (such as the posterior cingulate cortex) and their connectivity with a series of intrinsic circuits such as those related to attention, somato-motor, and fronto-parietal networks. These altered connectivity patterns predicted stuttering persistence (vs. recovery). Findings suggest that developmental alterations in the balance between functional neural networks (e.g., “lack of balance” in how attention processes regulate speech-motor control) may be important in relation to the development, appearance and/or maintenance of stuttering (Chang et al., 2018). In fact, based on this evidence, the DMN may interfere with the correct/timed recruitment of “goal-oriented” behaviors and TPNs, resulting clinically evident especially during speech and dysfluencies. In this regard, Garnett et al. (2022) found that PWS tend to exhibit heightened connectivity between the left superior temporal cortex (auditory regions) and regions of the DMN during “solo” speech tasks, again suggesting the existence of a possible interference exerted by the DMN during speech in PWS.

In this context, other than fMRI studies, useful evidence about resting-state neural activity may also be obtained by electroencephalography (EEG). In this case, available studies (Finitzo et al., 1991; Ozge et al., 2004; Joos et al., 2014; Saltuklaroglu et al., 2017) mainly suggest a tendency toward reduced power of the beta band (i.e., 13–30 Hz) in PWS. Furthermore, analysis of brain connectivity based on frequency bands indicates some anomalies of resting-state activity in DS (Joos et al., 2014; Ghaderi et al., 2018). Speculatively, the presence of an evident reduction in beta EEG power at rest in the brain of PWS could suggest the presence of reduced cerebral metabolism, possibly in relation to limitations in the supply of energy to neurons (e.g., sub-optimal glycolysis processes; Alm, 2021a).

In summary, some abnormalities related to (persistent) DS may be present well before the development of evident deficits in speech-motor programming and execution, likely influencing neural processing, and confirming that DS may be a more “general” disturbance than previously considered (i.e., not exclusively related to speech; Ludlow and Loucks, 2003; Busan et al., 2013, 2017). Importantly, as described in the next sections, these systems may be further influenced by the presence of added “neural noise,” such as that induced by excessive anxiety. A better comprehension of these mechanisms may furnish new suggestions for improved interventions in (persistent) DS.

## Interactions between atypical neural systems in DS and associated psychological/psychiatric characteristics: anxiety and stuttering

A common associated characteristic of (persistent) DS is the development of social anxiety, social phobia, and avoidance behaviors (Menzies et al., 1999; Iverach et al., 2011; Iverach and Rapee, 2014). PWS may also develop a stigmatized identity that contributes to avoidance, shame, and fear of speaking (Sisskin and Goldstein, 2022) which, in turn, might considerably impact their quality of life (Craig et al., 2009; Nang et al., 2018). It is now quite clear that an emotive/emotional difference is not the fundamental driving force behind development of stuttering in children (Reilly et al., 2009; Alm, 2014; Park et al., 2021; Delpeche et al., 2022). However, it is common that (persistent) stuttering results in association with some types of psychological profiles during school age or later (Iverach et al., 2009; St Clare et al., 2009; Iverach and Rapee, 2014). For example, social anxiety and unhelpful thoughts can develop into a major problem for PWS: these aspects have become the focus of specific treatment efforts, such as cognitive-behavioral therapy (Lowe et al., 2021), mindfulness (Emge and Pellowski, 2019), and, very recently, even computer/internet-based (Gunn et al., 2019: Menzies et al., 2019) and virtual reality approaches (Chard et al., 2023).

Accordingly, starting from the evidence of a more “vulnerable” speech-motor system in DS (Perkins et al., 1991; Ludlow and Loucks, 2003; Alm, 2004; Namasivayam and van Lieshout, 2011; Civier et al., 2013; Craig-McQuaide et al., 2014; Smith and Weber, 2017; Etchell et al., 2018; Busan, 2020; Chang and Guenther, 2020), Alm (2014) hypothesized that stuttering may be worsened by interference from “social cognition,” whereby the speaker’s perceptions of the listener or themselves burden an already vulnerable speech-motor control system, as a likely result of “maladaptive” processes. In fact, propositional (i.e., spontaneous) speech may be more affected in DS (as already suggested in Eisenson and Horowitz, 1945) and supported by regions such as the prefrontal cortex, supplementary motor area, basal ganglia, and the inferior frontal gyrus (see, for a perspective in DS, Chang and Guenther, 2020; Neef and Chang, 2024). However, a social-cognitive network (SCN, Figure 1C) including the medial prefrontal cortex and the anterior cingulate cortex (and, hence, also comprising regions of the DMN; adapted from Amodio and Frith, 2006; compare with Wu et al., 1995; Moran et al., 2006; Alm, 2014; Craig-McQuaide et al., 2014) is also present and mainly related to the processing of socially relevant information (in this case, perhaps also exchanging information with limbic regions; see Neef and Chang, 2024). A similar view is also proposed by other recent theories and visions about DS, supporting relevant suggestions about the effects of factors such as (inefficient) speech/language processing, monitoring, time pressure, physiological arousal, temperament, and/or emotional regulation (e.g., anxiety and fear) on speech-motor control systems (Perkins et al., 1991; Healey et al., 2004; Conture et al., 2006; Bloodstein and Ratner, 2008; Namasivayam and van Lieshout, 2011; Packman, 2012; Walden et al., 2012; Arenas, 2017; Smith and Weber, 2017). These processes also possibly involve (variable) changes in autonomic functions (see, for a brief review on DS, Garcia-Barrera and Davidow, 2015). Specifically, in the context of stuttering, social cognition may involve thoughts about oneself (e.g., negative evaluation) or concerns regarding the current speech situation, always in relation to main factors such as: (i) the importance or possible consequences of the situation; (ii) the possibility of stuttering; and (iii) the uncertainty about the best way to act (e.g., in order to avoid dysfluencies; Alm, 2014). Interestingly, this system also partially overlaps with regions of the salience network (SN, Figure 1D; Sridharan et al., 2008) that are useful for detecting or integrating sensorial (and emotional) stimuli, but also constituting a likely “switching” between DMN and networks useful for “externally-directed” cognition (Sridharan et al., 2008).

In this context, during “goal-directed” behavior (i.e., speech), the neural networks so far described are collaborating, trying to avoid (mutual) interferences (see, for a perspective on DS, Alm, 2014). As anticipated, the SN may facilitate the capacity to switch between the DMN (as already suggested, usually related to the resting condition and introspection) and TPNs such as the executive control networks (e.g., the central executive network, CEN; Gratton et al., 2018; Figure 1E). In fact, TPNs usually need attention that is focused on external cognitive or motor tasks (Corbetta and Shulman, 2002; Kincade et al., 2005), thus recruiting regions such as the insula and the frontal operculum, the supplementary motor cortex, the medial frontal lobe, the lateral premotor cortex, and the middle frontal gyrus, as well as the superior/inferior parietal lobule and posterior temporal gyri (Blumenfeld, 2016). Interestingly, most of these networks (i.e., SCN, DMN, and SN) have been suggested to have a role in negative reactions to stuttering, thus likely contributing to the development of social anxiety (Alm, 2014). As in a vicious circle, this anxiety is reinforced by the anticipation of stuttering which increases in extent and complexity throughout development (Jackson et al., 2015, 2018, 2019; Rodgers and Jackson, 2021), thus possibly resulting in further repetitive and negative thinking or rumination (Tichenor and Yaruss, 2020). Interestingly, Orpella et al. (2024) suggest that this may result in a further abnormal involvement of control on processes during speech production, especially prior to anticipated and stuttered (vs. fluent) speech (Jackson et al., 2022). In this regard, it has been suggested that internally-(vs. externally -)directed focus may contribute to disrupt simple/automatic movements (Wulf et al., 2001; McNevin et al., 2003; Kal et al., 2013), also in DS (e.g., Eichorn et al., 2016, 2019; Jackson et al., 2016).

A striking characteristic of stuttering is its typical variability in most cases, between situations and from moment to moment (Constantino et al., 2016; Tichenor and Yaruss, 2021), also as a function of audience (Jackson et al., 2016). In this context, Jackson et al. (2021) showed that, in DS, disfluencies are virtually eliminated during “private” speech (i.e., speaking “alone” condition). Again, this evidence relates with the capacity of PWS to anticipate their stuttering (Brocklehurst et al., 2013; Garcia-Barrera and Davidow, 2015; compare with Bloodstein, 1975; Arenas, 2017): conditions of stuttering anticipation are related to consistent overactivation of the right prefrontal cortex (Jackson et al., 2022). During “private” speech there would be no social evaluation, communicative aim, or the need to interpret listener information, thereby reducing potential interferences from the SCN/DMN on an already vulnerable speech-motor system (Alm, 2014, section 8.5; Smith and Weber, 2017). Thus, the difference between talking alone and talking to someone else appears to be sufficient to result in speech disruptions for PWS. Alm (2014) proposed that the SCN/DMN tends to interfere with the TPNs during social situations, thus triggering stuttering (a similar view of the role of DMN in DS was put forward in Chang et al., 2018). According to the proposed model, it can be expected that social anxiety and activation of the DMN will result in suppression of speech-motor related networks, thereby increasing the risk for stuttering (Walden et al., 2012; Brocklehurst et al., 2013; Alm, 2014; Smith and Weber, 2017).

In this regard, stuttering events seem to correlate with the presence of higher inhibitory activity in sensorimotor networks (Korzeczek et al., 2022; Orpella et al., 2024). In this case, evidence suggests that “maladaptive” processes, also involving the above-described patterns and, possibly, abnormal modulation of action-stopping networks (Neef et al., 2018a,^2022^, ^2023^; Busan et al., 2020; Korzeczek et al., 2022), may have a role in interaction with circuits involved in emotion regulation (Neef et al., 2018b,^2022^, ^2023^; Montag et al., 2019; Chow et al., 2023). Indeed, recent research (Yang et al., 2017; Toyomura et al., 2018) showed the presence of neurophysiological connections between emotional regulation and stuttering. For example, activity in the right amygdala (a key anatomical region in the limbic system involved in fear responses and emotions elaboration; Šimić et al., 2021) was correlated with disfluencies and discomfort in PWS, also influencing activity in the prefrontal cortex (which is part of an emotion-regulation system with the amygdala; Toyomura et al., 2018). Similarly, during a speech task, PWS showed increased functional connectivity of the right amygdala with prefrontal regions and the left insula (Yang et al., 2017). During resting-state conditions, PWS also showed stronger connectivity between the hippocampus and prefrontal/motor areas. In summary, the available evidence suggests that aberrant neural activity for anxiety regulation might have a role in the higher levels of anxiety observed in DS, possibly also in relation to stuttering severity. As a consequence, attention of PWS could be mainly “internally” focused (i.e., likely on the possible social consequences of stuttering), rather than being on the interlocutor.

All things considered, a potential rationale for the usage of psychedelics in DS will be developed in the next sections. There are several pre-clinical and clinical indications that psychedelics (e.g., LSD) may have a therapeutic role in alleviating anxiety (see, for a recent review, Inserra et al., 2023). Thus, by reducing anxiety in DS, psychedelics may modulate activity in overactivated (or underactivated) systems, thereby reducing difficulties and allowing “easier” control (i.e., with limited interferences) of speech-motor systems (Alm, 2014; Jackson et al., 2022; Orpella et al., 2024). The mechanisms could be through effects on brain metabolism and their capacity to promote plasticity, also in networks such as the SCN/DMN and emotional regulatory systems (compare with Ly et al., 2018).

## Psychedelic drugs: possible brain mechanisms

Drugs producing psychedelic experiences are mainly known to act via 5-HT2A receptor agonism, even if the agonism and/or partial agonism of receptors such as 5-HT1A, 5-HT7, and D2 also play an important role (Inserra et al., 2021a; Ling et al., 2022). 5-HT2A receptor agonism indirectly induces glutamate release, for example by activating AMPA receptors (Aghajanian and Marek, 1997; De Gregorio et al., 2021). The activation of AMPA and glutamatergic systems is likely linked to the neuronal plasticity of brain regions such as the hippocampus, amygdala, and prefrontal cortex, even if more studies are needed to demonstrate a direct link (Ly et al., 2018; Inserra et al., 2021a). It has been recently suggested that the effects of this class of drugs on neurotrophic signals and plasticity may also depend on the binding of the brain-derived neurotrophic factor (BDNF) receptor TrkB, which is useful in promoting endogenous BDNF signaling (see Moliner et al., 2023). However, since antidepressants drugs (e.g., selective serotonin reuptake inhibitors) also increase BDNF (even if psychedelics would have higher affinity), it is difficult to understand if this could be specific to psychedelics or a common mechanism for antidepressant drugs (Saarelainen et al., 2003). At regular/high doses, psychedelics produce an experience with vivid hallucinations and mind alteration, and may also induce profound psychological and “mystical” experiences (Griffiths et al., 2008; Hirota and Lambert, 2022; Vamvakopoulou et al., 2023). In this context, psychedelic drugs such as psilocybin may elicit a subjective state that could be described as “a waking dream,” where memory formation and insights usually remain intact, but the nature and range of consciously experienced mental processes are broad and shifted toward “imprecision” (relative to normal consciousness). More specifically, sensory distortions, strong emotions, phantasmagoria, and blurring of conceptual boundaries are common and dose-dependent. Sensations of “bliss,” “elemental imagery,” “experience of unity,” “spiritual experience,” “insightfulness,” “audio-visual synesthesia,” and others are also usually very frequently reported under psilocybin. However, since psychedelic drugs may also be viewed as non-specific amplifiers of mental processes, the range and degree of reported experiences also includes negative ones such as panic, paranoia, and hallucinations (Rucker and Erritzoe, 2023). In this context, the safety of psychedelics is contingent upon dosage, with psychological adverse events usually predominating over physiological ones. The pharmacokinetics (PK) of psychedelics is complex, and each drug has a distinct profile. For example, LSD (200 μg) has a plasma half-life of 2.5 h, but the hallucinogenic effect is long-lasting (about 12 h; see Dolder et al., 2017). On the other hand, psilocybin is rapidly converted in psilocin after oral ingestion by plasmatic and hepatic enzymes, and elimination half-life is about 1.4 h. On the other hand, DMT is not bio-available and should be administered in humans by slow intravenous infusion, obtaining rapid attainment of peak plasma concentrations followed by rapid clearance (Good et al., 2023). When considering the pharmacodynamics (PD) of psychedelics, it is not directly associated with PK: for example, for LSD, the effects on anxiety are detectable after a few days and may persist for weeks (Holze et al., 2023). This peculiar PD is likely related to neuroplastic and/or epigenetic modulations induced by psychedelics and, thus, to their effects on neural circuits (Inserra et al., 2021a).

When combined with psychological support/therapy (Fuentes et al., 2020; Gobbi et al., 2022; Lowe et al., 2022), the therapeutic effects of psychedelics for mental diseases like major and resistant depression (Carhart-Harris et al., 2021; Goodwin et al., 2022), anxiety (Gasser et al., 2014, 2015; Holze et al., 2023), and alcohol use disorder (Bogenschutz et al., 2022) are highly promising, even if one cannot rule out the essential role of psychotherapy in the reappraisal of negative biases after the psychedelic experience. Undoubtedly, more studies are required to understand these links. Consistently, in animals it has been demonstrated that psychedelics, such as LSD, may reduce stress-induced anxiety through serotonergic mechanisms: during stress, the firing activity of 5-HT neurons in the dorsal raphe is decreased, but chronic administration of LSD may restore a more physiological activity through desensitization of 5-HT1A auto-receptors (De Gregorio et al., 2022). Moreover, LSD treatment (i.e., 30 μg/kg once a day for 7 days) allows spinogenesis to increase (see De Gregorio et al., 2021, 2022), and, in parallel, promotes the methylation of genes involved in neuroplasticity processes of brain regions such as the prefrontal cortex (Inserra et al., 2022). However, further studies are required to better understand if epigenetic processes like hyper-methylation/hypo-methylation are directly linked to spinogenesis, or if spinogenesis is independent from epigenetic mechanisms. Interestingly, it has also been proposed that psychedelics can evoke “metaplasticity,” a phenomenon making neurons more susceptible to stimuli that induce plasticity (e.g., hormones, neurotrophic factors, etc.; Nardou et al., 2023): this is compelling for DS, since it may involve reshaping of neurons and networks that may be dysfunctional in stuttering.

In this context, more work is needed to understand how the increased neural plasticity induced by psychedelics (see Calder and Hasler, 2023) is linked to the changes in brain circuitry and increase in “entropy” of neural networks (Carhart-Harris and Friston, 2019; Inserra et al., 2021a; Herzog et al., 2023; Ort et al., 2023; Wall et al., 2023). The “acute” and “rebound” activity could be in opposite directions when considering brain metabolism (see Vollenweider et al., 1997; Carhart-Harris et al., 2012). For example, psilocybin seems to result in a rapid decrease in activity of hub regions (such as the thalamus or the anterior/posterior cingulate regions; Carhart-Harris et al., 2012), possibly followed in time by an increase in brain metabolism of frontal regions (Vollenweider et al., 1997; see Lewis et al., 2017, for a discussion combining “hyper-frontal” hypotheses of psilocybin with studies demonstrating a more general decrease in brain perfusion). Moreover, psychedelics seem to “disrupt” connectivity, especially in resting-state networks such as the DMN, also resulting in induced modifications of the functional connectivity between these circuits and other brain networks (Carhart-Harris et al., 2013, 2017; Daws et al., 2022; Felsch and Kuypers, 2022) that, for example, may favor “goal-directed” behaviors. In this context, a series of different models have been proposed that attempt to explain the various effects of doses of psychedelic drugs on the brain, also making assumptions about their induced neural modulations (e.g., DMN; Carhart-Harris and Friston, 2019; Vollenweider and Preller, 2020; Daws et al., 2022; Doss et al., 2022; Gattuso et al., 2023; Herzog et al., 2023; Wall et al., 2023). Nonetheless, it is still not clear what is the main brain target of the therapeutic potential of, for example, psilocybin (Gattuso et al., 2023). A recent systematic review by Gattuso et al. (2023) constituted further evidence in favor of the idea that the DMN may be linked to effects such as “ego dissolution,” enhanced mental health, and wellbeing (Carhart-Harris et al., 2014; Nour et al., 2016). In this context, recent studies indicate that the effects may be related to the modulation of functional connectivity between networks, rather than within them (see Gattuso et al., 2023; compare with Tagliazucchi et al., 2016; Girn et al., 2022). This can mean that brain areas which typically have strong functional connections may be not further and mutually reinforced but, on the other hand, brain areas whose activity is only “weakly” correlated may tend to become more connected (see Gattuso et al., 2023; compare with Tagliazucchi et al., 2016; Girn et al., 2022). Some evidence suggests that diffuse effects (i.e., up-regulation of c-FOS, a marker of neuroplasticity processes) on sensorimotor circuits and association regions, as well as on anterior cingulate cortex and sub-cortical regions is present (such as the thalamus and the amygdala; see Davoudian et al., 2023; compare with Calder and Hasler, 2023). In particular, sensorial or “gating” structures, such as the thalamus (or associated structures, such as the claustrum), may play an important role in these processes, especially when considering their diffuse cortical connections that mainly express 5-HT2A receptors (Tagliazucchi et al., 2016; Doss et al., 2022; compare with Weber and Andrade, 2010). In support of this, it has been recently reported that LSD modulates activity in thalamic nuclei projecting to the prefrontal cortex, thus likely contributing to alteration of consciousness functions (Inserra et al., 2021b). In addition, Timmermann et al. (2023) reported that psychedelics may favor a “global hyper-connectivity,” also collapsing hierarchical organization and reducing intra-network integrity. By means of fMRI/EEG co-registration, they demonstrated that increased “entropy” correlated with decreased alpha power (compare with Annerud Awrohum, 2021), and also showed that regions with the densest expression of 5-HT2A receptors (PET data) were the most affected by the psychedelic compound (Timmermann et al., 2023). In this regard, animal models suggest that the effect of psychedelics (e.g., LSD) may be mediated by brain regions and networks that are strongly involved in the control of anxiety (DMN, prefrontal cortex-amygdala circuits and cortico-striato-thalamo-cortical networks; Inserra et al., 2023). Even if more translational research is required to understand the exact mechanisms involved, several studies have demonstrated the anti-anxiety effects of LSD in animals, as well as also reported in clinical trials (compare with Holze et al., 2023). Also, LSD increases social behavior and decreases social anxiety, likely through a prefrontal cortex-mediated effect on glutamatergic neurons, which is paralleled by an increase in mTOR phosphorylation (De Gregorio et al., 2022).

In this context, when considering conditions such as social anxiety disorder (SAD, also characterized by aberrant functional connectivity of the SN/DMN systems; Felsch and Kuypers, 2022) and the possibility to “drive” the effects of psychedelics with assisted intervention (e.g., psychotherapy; The Lancet Regional Health - Europe, 2023), evidence suggests that treatments based on psilocybin-assisted meditation might result in clinical changes modulating connectivity of these networks, thus increasing control over amygdala “reactivity” (Felsch and Kuypers, 2022; compare with Carhart-Harris et al., 2017). In fact, SAD is usually characterized by aberrant patterns of brain activity in the amygdala, also comprising regions such as the temporal cortex, insula, basal nuclei, prefrontal, and cingulate regions (Felsch and Kuypers, 2022). This can be the consequence of pathophysiological and/or maladaptive mechanisms. Evidence of abnormalities in serotonergic and dopaminergic circuits has also been described (Hjorth et al., 2021; Felsch and Kuypers, 2022). As already reported, a common associated characteristic of DS is the development of anxiety, social phobia, and avoidance behaviors (Menzies et al., 1999; Iverach et al., 2011; Iverach and Rapee, 2014). In summary, the possible positive effects of psychedelics on (social) anxiety reinforce the rationale of their utilization in DS.

## A possible therapeutic rationale for psychedelics in DS

The previous sections described the neuropathological mechanisms of DS, as well as the likely mechanisms for psychedelic drugs to act on brain networks. Considering that (frontal) brain metabolism may be aberrant in PWS, and that the “maladaptive” intervention of networks such as the SCN and the DMN could add further “neural noise” to the system (thus worsening the functionality of “goal-directed” networks and behaviors; compare with Alm, 2014; Jackson et al., 2022; Orpella et al., 2024), we propose that controlled treatment with psychedelics should be investigated to help in disrupting maladaptive neural functions in DS, thus restoring a more “adaptive” plasticity in the neural networks involved. More specifically, this should be done in the attempt to reduce social anxiety interacting with speech-motor networks, both precipitating and maintaining stuttering [always keeping in mind that serotonergic drugs may also modulate the functioning of motor excitability in humans (Thorstensen et al., 2024), as well as speech-motor circuits in DS (see Busan et al., 2009)].

This becomes especially significant when considering that, as described above, (persistent) DS is normally associated with a series of associated features and effects, including negative self-perception, negative perception by others, social phobia, avoidance behaviors, anxiety, and depression (Iverach et al., 2011; Iverach and Rapee, 2014; Sander and Osborne, 2019). Indeed, in agreement with a growing body of data, a considerable prevalence of “social” anxiety is evident among PWS (Kraaimaat et al., 2002; Blumgart et al., 2010; Iverach and Rapee, 2014; Iverach et al., 2017), with the clear possibility that these aspects may also act in significantly worsening speech-motor performance and fluency (compare with Alm, 2014; Jackson et al., 2021, 2022; Orpella et al., 2024). A variety of interconnected elements, including the fear of being negatively evaluated (i.e., negative social cognition) and attentional biases (e.g., elevated “self-focused” attention), as well as “anticipatory” and/or “post-event” processing, may have an impact on the development and maintenance of dysfluencies and social anxiety in PWS (see Alm, 2014; Iverach et al., 2017). As mentioned above, stuttering anticipation may be strongly related to overactivation of the right prefrontal cortex (Jackson et al., 2022), thus likely deriving from (or influencing) neural interactions among the DMN, SCN, SN, and TPNs. In fact, excessive involvement of the DMN has been reported to have a role in conditions of psychological ill-health, such as depression and social anxiety (Hamilton et al., 2011). For example, in individuals with major depressive disorder, this may represent the neural substrate for experiencing higher levels of maladaptive rumination (Hamilton et al., 2011). Accordingly, studies of psilocybin have shown promising results in these disorders (Lowe et al., 2022), and reduced overactivation of the DMN could be a key factor (Smigielski et al., 2019; Aday et al., 2020; Gattuso et al., 2023). In this context, since it has also been demonstrated that, for example, LSD can improve social behavior (De Gregorio et al., 2021) and stress-induced anxiety (De Gregorio et al., 2022), psychedelics could act on DS by improving social anxiety and generalized anxiety. Indeed, a repeated LSD regimen increases social behavior in mice by activating glutamatergic neurons in the prefrontal cortex, potentiating synaptic responses of 5-HT2A and AMPA receptors (De Gregorio et al., 2021), as well as reversing the 5-HT firing deficiency induced by stress, thus alleviating anxiety (De Gregorio et al., 2022).

When considering emotion encoding and processing, the amygdala is a key anatomical region in the limbic system (Šimić et al., 2021) and it has been demonstrated that classic psychedelics can modulate its responsiveness (Kraehenmann et al., 2015; Carhart-Harris et al., 2017; Mueller et al., 2017; Roseman et al., 2018). In general, increased amygdala activation may be a neurophysiologic response that could be considered as a “neural marker” of pathological conditions such as PTSD (Francati et al., 2007). Despite the limited evidence regarding the involvement of the limbic system in stuttering, recent studies have sustained the role of amygdala activation in persistent DS (Yang et al., 2017; Toyomura et al., 2018). For example, amygdala was found to significantly correlate with occurrence of disfluencies in PWS (Toyomura et al., 2018). Interestingly, psychedelics have been demonstrated to induce dynamic structural/functional plastic modulations in “fear-extinction” neural circuits, thus tempering/modulating the amygdala’s hyper-reactivity to threat-related stimuli (Glavonic et al., 2022). Similarly, changes in amygdala and prefrontal functional connectivity have been reported during emotional processing after administration of psilocybin for treatment-resistant depression (Mertens et al., 2020). Psychedelic-induced plasticity may also need the recruitment of other structures, such as astrocytes, to promote their effects (Martin and Nichols, 2016). Interestingly, as mentioned above, astrocytes may also play a role in the impaired neural metabolism related to DS (Maguire et al., 2021; Turk et al., 2021). In this context, Maguire et al. (2021) showed that the positive effects of risperidone on stuttering may be the consequence of an increase in brain metabolism, possibly mediated by astrocytes.

In summary, a range of evidence from the use of psychedelics [in various conditions which may or not share some features with DS -e.g., characteristics of SAD and PTSD; compare, for example, with Etkin and Wager (2007), Francati et al. (2007), Felsch and Kuypers (2022)] supports the theoretical foundations to test the impact of this class of drugs on DS. In addition, Jackson et al. (2024) conducted a qualitative analysis to explore potential benefits and negative effects of psychedelics on stuttering, starting from anecdotal evidence of PWS using psychedelics for recreational purposes. About 75% of subjects reported some overall positive effects. More specifically, 60% of participants indicated positive behavioral changes such as reduced stuttering and increased control of speech; 40% reported positive emotional benefits, 15% reported some positive cognitive changes, and 7% indicated some additional positive social effects. On the other hand, about 10% of participants reported some negative behavioral effects, such as increased stuttering or reduced speech control. Moreover, there is a very recent case-report of resolution of stuttering during treatment with ketamine (Bolton et al., 2023; ketamine is considered a psychedelic drug, even if not part of the classic psychedelics group; compare with Aleksandrova and Phillips, 2021). In this case, a 60-year-old woman who had been a lifelong stutterer was prescribed ketamine for an unrelated condition (i.e., depression), and experienced an almost immediate resolution of stuttering. All considered, and also bearing in mind their fast anxiolytic component (Dos Santos et al., 2016), this evidence might call for the possible evaluation of the effects of psychedelics in the context of persistent DS in adulthood. For example, since trauma-informed therapies are useful for some PWS, incorporating psychedelic treatment may be worth exploring in stuttering.

## Conclusion

DS still represents an unsolved and often underestimated health challenge in terms of its impact on overall quality of life (Craig et al., 2009). Thus, research oriented to investigate new possible therapeutic agents is needed, particularly given the often limited or unsustainable effects of current behavioral interventions (Nye et al., 2013). Paradoxically, some of the previously tested pharmacological substances known to lessen stuttering in some persons have also been found able to “induce” it in others (i.e., drug-induced stuttering; Brady, 1998; Alm, 2004; Ekhart et al., 2021), likely due to its complex and not yet fully defined etiology, as well as to possible high inter-patient variability (Brady, 1991; Alm, 2004). In this regard, randomized clinical trials (RCTs) focusing on drug interventions for persistent stuttering have shown mixed results (Maguire et al., 2020). In summary, the complexity of stuttering (e.g., with potential sub-types and varying responses to treatments) makes it challenging to draw definitive conclusions.

As a consequence, DS might benefit from further exploring the potential of clinical investigations of psychedelics in (adult) PWS. To date (to the best of our knowledge), there are no registered and/or completed RCTs investigating the possible efficacy of this class of drugs in DS (apart from anecdotal evidence; Bolton et al., 2023; Jackson et al., 2024). In this regard, prospective, randomized, blinded, and controlled trials will be required to understand the efficacy and safety of psychedelic therapies for the treatment of DS. More specifically, the design of such trials will need to answer questions such as: (i) which compounds should be considered, thus defining optimal doses, possible effects, and duration of therapy; (ii) whether repetitive micro-dosing or bolus-dosing will lead to better outcomes; (iii) the main inclusion/exclusion criteria for treatment, as well as the outcomes and measures that should be considered to demonstrate efficacy; (iv) if association with guided psychotherapy (or speech therapy) will lead to better outcomes; (v) if psychedelics could be an option for any person who stutters. From a methodological point of view, we can anticipate that RCTs evaluating psychedelics in DS should consider the effects on stuttering severity and social anxiety as primary outcomes, perhaps also considering secondary outcomes such as communication attitudes and/or evaluation of neurophysiological underpinnings (e.g., pre- vs. post-treatment functional connectivity of the brain, measured by fMRI and/or EEG). In this context, we can hypothesize that psychedelic treatment would be able to support higher “energy” resources to impaired speech-motor circuits in DS (thus, improving the stuttering “trait”), likely resulting in more “focused” neural activity during “goal-directed” behavior (e.g., propositional speech). This should also allow for lower levels of social anxiety and improved speech fluency in PWS (thus, improving the stuttering “state”).

The evidence suggests that, if correctly administered, psychedelics may be useful and safe (also considering secondary effects -e.g., hallucinogenic ones-; Dos Santos et al., 2018; Schlag et al., 2022; compare with Holze et al., 2022a,b), helping to treat and better understand brain (network) anomalies related to (psychiatric) disorders. When considering the risk for negative effects, settings and support during sessions are essential (Aday et al., 2020). It has been reported that almost a third of participants experience acute anxiety at some point during sessions with high dose, while, on the other hand, it has also been shown that no long-term negative effects occurred in studies involving more than 2,000 persons (see Ross et al., 2016; Aday et al., 2020).

In this context, the landscape surrounding psychedelic research and therapy is rapidly evolving, with increasing interest and recognition of their therapeutic potential. However, navigating the legal and ethical complexities remains a significant challenge for researchers, clinicians, and policymakers alike. In 2024, the decision to administer psychedelics nationally or within a specific state in the United States is largely determined by regulatory bodies such as the Food and Drug Administration (FDA) at the federal level as well as state-level legislative bodies. The FDA oversees the approval process for clinical trials involving psychedelics and other drugs, ensuring that they meet standards for safety and efficacy. However, individual states may also have their own regulations governing the use of psychedelics in research or therapeutic settings. For example, Oregon has legalized the sales of psychedelics, while in Colorado the use of psilocybin is allowed only in psychiatric settings. Similarly, in June 2023, Australia legalized the use of these drugs by psychiatrists (see Haridy, 2023). Outside the US, regulations vary significantly from country to country.^1^ In some nations, psychedelics may be strictly prohibited, while in others they may be legal for certain therapeutic purposes or subject to medical research under specific regulations. For example, Canada has permitted the use of psilocybin in a context of special access programs, where psychiatrists or physicians can make a request in case of treatment-resistant depression, PTSD, or end-of-life anxiety.

In conclusion, we suggest that, starting from observational data collected from PWS who self-medicate stuttering with psychedelics (Bolton et al., 2023; Jackson et al., 2024), their effects could be also investigated more in depth in the context of an underestimated speech-motor fluency disorder that strongly impairs quality of life of people with DS, thus contributing to boost and improve outcomes of currently available interventions (e.g., speech therapy). Currently, our team is actively trying to progress toward the obtaining of funding for conducting sponsored RCTs, drawing upon the scientific rationale outlined herein. This initiative represents a concerted effort to translate theoretical hypotheses into practical/empirical research.

## Data availability statement

The original contributions presented in this study are included in this article/supplementary material, further inquiries can be directed to the corresponding author.

## Author contributions

GP: Conceptualization, Formal analysis, Investigation, Project administration, Supervision, Writing – original draft, Writing – review & editing. PB: Formal analysis, Funding acquisition, Investigation, Resources, Supervision, Visualization, Writing – review & editing. EJ: Writing – review & editing. PA: Writing – review & editing. DD: Writing – review & editing. GM: Resources, Writing – review & editing. GuG: Supervision, Writing – review & editing. GaG: Validation, Writing – review & editing. DE: Writing – review & editing. RC-H: Supervision, Validation, Writing – review & editing.

## Footnotes

1
https://psychedelicalpha.com/data/worldwide-psychedelic-laws
